# An empirical study of the effect of a flooding event caused by extreme rainfall on preventive behaviors against COVID-19

**DOI:** 10.3389/fpubh.2022.1003362

**Published:** 2022-09-29

**Authors:** Chengcheng Liu, Qibin Lu, Qiang Zhang

**Affiliations:** ^1^School of Social Development and Public Policy, Beijing Normal University, Beijing, China; ^2^Centre of Emergency Management and Humanitarian Action, International Academy of the Red Cross and Red Crescent, Suzhou, China; ^3^Community Safety Committee, China Society of Emergency Management (CSEM), Beijing, China; ^4^Center for Crisis Management Research, Tsinghua University, Beijing, China

**Keywords:** the COVID-19, preventive behaviors, flood risk perception, response to flood risk, social capital, community disaster preparedness

## Abstract

Since the outbreak of COVID-19, wearing masks, vaccinations, and maintaining a safe distance has become social behaviors advocated by the government and widely adopted by the public. At the same time, unpredictable natural disaster risks brought by extreme climate change compound difficulties during epidemics and cause systemic risks that influence the existing pattern of epidemic prevention. Therefore, it is necessary to explore the effect of natural disaster risk caused by climate change on the response to outbreaks in the context of the COVID-19 epidemic. This study will focus on individual-level epidemic prevention behaviors, taking as an example the significant risk of severe destructive flooding caused by heavy rains in Henan, China, on July 20, 2021, which claimed 398 lives, to explore the effect of floods on the preventive behaviors of residents in the hardest hit areas against COVID-19. Through the multi-stage stratified random sampling of the affected residents in Zhengzhou, Xinxiang, Hebi, Luoyang, Anyang, and other cities in Henan Province, 2,744 affected people were surveyed via questionnaires. Through the linear regression model and moderating effect analysis, the study found that after floods, the individual's flood risk perception and response behaviors significantly correlated with the individual's prevention behaviors against COVID-19. Specifically, both flood risk perception and response behaviors strengthened the individual's prevention behaviors. Furthermore, the study also found that community risk preparation behavior and social capital can moderate the above relationship to a certain extent. The research can guide risk communication under the compound risk scenario and prevent risky public behavior under the consistent presence of COVID-19 in the community.

## Introduction

The COVID-19 pandemic is going into its third year and is expected to be a protracted public health crisis (1). As of July 2022, confirmed cases of COVID-19 have surpassed 565 million, with over 6 million deaths reported to the World Health Organization (WHO)^1^. To curtail the spread, individual prevention actions such as wearing masks, receiving vaccinations, and maintaining a safe distance were particularly central to government policies and widely adopted by the public (2, 3). However, at the same time, unpredictable natural disaster risks brought by extreme climate change are increasing in frequency and intensity, which makes them more likely to collide with the COVID-19 pandemic and impact the existing pattern of epidemic prevention and public response (4). For example, measures to safeguard populations from floods—timely evacuation and congregate sheltering procedures—may elevate the risk for COVID-19 transmission since those actions encourage people to share crowded spaces and run counter to COVID-19 mitigation measures such as physical distancing (5).

Moreover, the unpredictable natural disaster may be distracting and overshadow the public reckoning with COVID-19. Based on the consideration above, the current study intends to take as an example the severe destructive flooding caused by heavy rains in Henan, China, on July 20, 2021, to explore the effect of natural disasters on preventive behaviors against COVID-19. Several implications from these findings could be used for risk communication and emergency management to limit the effects of multiple hazard risks.

Risk perception refers to the comprehensive evaluation of perceived probability and perceived consequences (6), which has emerged as the basis for exploring populations' response to hazards (7, 8). This means that people must first perceive risk threats to them before considering or adopting protective behaviors (9), which has been emphasized in many research frameworks of disaster, such as protection motivation theory (PMT) (10–12), the motivation intention volition model (MIV) (6, 13), the theory of planned behavior (TPB) (14, 15), the theory of reasoned action (TRA) (16, 17), etc. Individuals can further evaluate the overall threat and their coping behaviors through risk perception, which can determine their risk-response strategies (9). However, most studies that link risk perception with the public response have focused on a single type of disaster, such as earthquakes (18), floods (19), or COVID-19 (17), and few have been applied to multiple disaster settings (16, 20). In the multi-hazard context, different hazards could stimulate different levels of risk perception (21), which can have an intertwined effect on different disaster-response strategies. As pointed out by Botzen, the perception of the COVID-19 pandemic had an opposite effect on evacuation intention during the subsequent hurricane season (22). To better recognize how the public responds to COVID-19 when overlapped with the flood, the current study examined the relationship between flood risk perception and preventive behaviors for COVID-19.

According to the risk perception paradox, the correlation between risk perception and protective measures against hazards is not necessarily tenable (23), whereas exploring the influence of the response to flood risk on COVID-19 preventive behaviors may be another potential path to explore the reinforcing/substituting effect of natural disasters on preventive behaviors against COVID-19. There are several reasons for this hypothesis. The protective action decision model (PADM), a circular model for explaining the process of action decisions (24), highlights that individual behavioral responses could act as social cues to initiate further a series of decisions, which provides theoretical evidence for exploring the influence of flood behavioral responses on preventive behaviors against COVID-19. In addition, several studies suggest the finite pool of worry effect, indicating that individuals have limited resources, and when focusing on one threat, attention to other risks decreases (25). Thus, concerns over COVID-19 may be reduced after experiencing sudden flooding.

Moreover, when a disaster hits, communities are the actual first responders (26), especially in providing immediate life-saving assistance (27). Therefore, when conducting disaster-related studies, it is crucial to explore the role of the community context to which individuals belong from a socioecological perspective (26). As described below, community context could be an external environmental factor to moderate the correlations between flood risk perception and COVID-19 preventive behaviors and between flood response and COVID-19 preventive behaviors.

First, social capital has increasingly drawn attention in disaster-related studies, partly because it touches on the heart of the therapeutic community under extreme events (28). Generally, social capital refers to social cohesion and personal investment in communities (29) and contains core components such as trust, reciprocity, norms, etc (30). Compared to physical or human capital, social capital is the least damaged and can be renewed/enhanced during disasters (31), which is crucial for improving community resilience during a disaster (28). For example, prior research regarding typhoons and heavy rain in Korea suggested that communities with high social capital, specifically civic engagement and trust, tended to respond to disasters better (32). In addition, social capital has been linked to risk perception in disaster-related studies (33, 34). Philipp Babcicky and his colleague stated that social capital harms risk perception since individuals who perceive their social context as supportive tend to judge themselves at lower disaster risk (33). However, little is known about how social capital is linked to the multi-hazard context. Specifically, for our research questions, we seek to examine the moderating role of social capitals on the associations between risk perception and preventive behaviors against disasters, and between flood response behaviors and COVID-19 preventive behaviors, especially in the multi-hazard context during the pandemic.

Similarly, attention to community disaster preparedness, including its role and significance in disaster management, continues to grow. Community-based disaster preparedness has been recognized as a critical element in disaster prevention (35). The literature highlighted that community-level preparedness could powerfully increase individual capacity to counter risk (36). Cuba's low disaster casualty rate during the hurricane season was one of the examples that benefited from community disaster preparedness in advance, including emergency knowledge training and community drills (37). In addition, community disaster preparedness has been confirmed to correlate with risk perception significantly (38). Specifically, individuals with high perception are more likely to perceive themselves as more vulnerable to disasters (39), which could further encourage them to engage in community preparedness (40). Again, however, few studies have combined community disaster preparedness, risk perception, and individual risk response to explore the underlying mechanism between these factors in the multi-hazard context. Our research explores the moderating role of community disaster preparedness on the associations between flood risk perception and preventive behaviors against COVID-19 and between flood response behaviors and COVID-19 preventive behaviors.

Overall, the primary purposes of this research are: (1) exploring the effect of floods (including flood risk perception and response behaviors) on the COVID-19 prevention behaviors among residents in the hardest-hit areas after the severe destructive flooding, and (2) examining the moderating effect of community context, specifically social capital and community disaster preparedness, on the above relationship. The corresponding findings can improve risk communication and disaster mitigation activities.

## Materials and methods

### Data collection and sampling

On July 20, 2021, an unprecedented meteorological event struck Zhengzhou in central China's Henan Province. The rainfall volume broke the historical record in mainland China and caused destructive flooding in Zhengzhou and its nearby areas (41). The rainstorms and flood disasters caused 398 deaths and direct economic losses of more than 120 billion RMB. A cross-sectional and a multi-stage stratified random sampling survey was conducted in August 2021 to explore the public response to flooding and COVID-19. The survey was in the form of online questionnaire and questionnaires were distributed to Zhengzhou, Hebi, Xinxiang, Anyang, Luoyang, etc., which were all the worst-hit areas. A total of 3,000 participants in 150 communities were invited to participate in the survey. Participation was voluntary, and written informed consent was received before responding to the questionnaire. In total, 2,744 respondents completed the questionnaires, and the response rate was 91.47%. Before data processing, 461 questionnaires were discarded due to missing values. Ultimately, 2,283 valid samples were included for further analyses. The School of Social Development and Public Policy of Beijing Normal University approved our study.

### Measurement

#### Response to flooding risk

Referring to previous studies on flood protective behaviors, the response to the flood risk was measured by three items: flood information collection, emergency evacuation (42), and volunteer participation (43). Every item had been measured by the same question “When the flood struck, did you take this countermeasure?.” Participants were required to answer the question on a scale of “yes-1 point” or “no-0 points.”

#### Flood risk perception

Studies on disaster risk perception are grounded in cognitive psychology, which defined and measured risk perception by the integrated evaluation of the perceived probability and perceived consequences of the exact disaster event (6, 19). Hence, we asked participants, “What is the probability for you to encounter such an event in your place?” and “To what extent does such an incident affect you negatively?” to examine the public flood risk perception. Both questions were answered using a 5-point Likert scale. Ultimately, risk perception equals the evaluation of the perceived probability multiplied by the perceived consequences. The total risk perception score ranged from 0 to 25, with a higher score indicating a higher risk perception of floods.

#### COVID-19 preventive behaviors

Based on the above discussion, the assessment of individuals' COVID-19 preventive response was composed of 4 behaviors: COVID-19 vaccination (vaccinated/unvaccinated), wearing a mask when going out (yes/no), reminding others to wear a mask (yes/no), and reminding others of social distancing (yes/no). All questions were answered by “yes-1 point” or “no-0 points.”

#### Community's social capital

The literature suggests that components such as civic engagement, norms of reciprocity, trust, and belief are all components of social capital (30, 44, 45). Correspondingly, the measurement of social capital in the current study contained elements such as trust, mutual assistance, community/village affairs participation, contact with community/village officials, community/village service equity, etc. The participants were asked to answer each item using a 5-point Likert scale. Moreover, Cronbach's alpha coefficient of the social capital questionnaire was 0.82, denoting acceptable internal consistency (46). Furthermore, we performed factor analysis to measure each community's social capital level, and the Kaiser–Meyer–Olkin (KMO) test value was good at 0.80, given its requirement to exceed 0.60 (47). In addition, Bartlett's test of sphericity was significant. Namely, the *P*-value (0.000) was <0.05 (48, 49).

#### Community disaster preparedness

As a primary element of community resilience, community preparedness was conceptualized as the capacity of the community to prepare for disasters in the short and long term (50). Based on the literature on community preparedness (51–53), emergency plans, emergency knowledge, hazard maps of communities/villages, emergency evacuation drills, community emergency response teams, and other protective activities were included in the current study. Several items had a limited range of responses (“yes,” “no,” or “don't know”) (53).

#### Potential confounding variables

In terms of the variables associated with risk-response behaviors, a few studies have focused on the effect of sociodemographic factors, such as gender (male/female), age, years of education (54), marital status (unmarried/married/widowed or divorced) (55), workplace (in-county/out-of-county) (56) and satisfaction with income (very dissatisfied/partially dissatisfied/general/partially satisfied/very satisfied) (57). In this context, disaster experience (54), agricultural insurance participation status (insured/uninsured) (58), and membership status with the community management committee/village committee (yes/no) (59) were also included in the further analyses.

### Statistical analyses

Data analyses were conducted with Stata. First, frequencies or mean values of COVID-19 preventive behaviors, risk perception and response to flood risk, social capital, community preparedness for flood risk, sociodemographic variables, etc., were described. Furthermore, the correlation matrix model was used to identify variables associated with COVID-19 preventive behaviors. Linear regression analyses using all the potential confounding variables as independent variables and COVID-19 preventive behaviors as outcome variables were conducted to identify the relationships among flood risk perception, response, and COVID-19 preventive behaviors. According to the findings of the linear regression models, the moderating effects of social capital and community preparedness for flood risk on the association between flood risk perception and COVID-19 preventive behaviors were examined. Additionally, the current study explores the moderating effects of social capital and community preparedness for flood risk on the association between flood response and COVID-19 preventive behaviors. Standardized regression coefficients (beta) and their *P* values were used to quantify the relationships between variables and COVID-19 preventive behaviors. The significance level was set at *P* < 0.05.

## Results

### Descriptive analysis

A total of 2,283 respondents met our criteria, and their COVID-19 preventive behaviors, flood risk perception and response, social capital, community preparedness for flood risk, and demographic characteristics are shown in Table 1. Among the total sample, the average number of COVID-19 preventive behaviors was 3.49, indicating that the public adopted better COVID-19 preventive behaviors when overlapped with the flood. The mean flood risk perception and response behavior scores were 12.30 and 2.52, respectively. Regarding social capital, more than 70% of respondents reported that most people in their community/village were trustworthy. The percentage of mutual assistance reached 82.21%. Nearly 90% of respondents reported their willingness to participate in community/village affairs. Moreover, the proportion of individuals with close contact with community/village officials was 60.98%. A total of 71.26% of participants believed that their community/village's services were fair or very fair. In terms of community preparedness for flood risk, the average level was 1.94, which was below its median.

**Table 1:** Descriptive analysis (*N* = 2,283).

**Variable**	**Frequency**	**Percent (%)**
**Gender**		
Female	897	39.29
Male	1,386	60.71
**Marital status**		
Unmarried	228	9.99
Married	1,984	86.90
Divorced/widowed	71	3.11
**Member of the community management committee/village committee**		
Yes	287	12.57
No	1,996	87.43
**Workplace**		
In-county	1,642	71.92
Out-of-county	641	28.08
**Agricultural insurance participation status**		
Uninsured	2,036	89.18
Insured	247	10.82
**Disaster experience**		
Yes	247	10.82
No	2,036	89.18
**Satisfaction with income**		
Very dissatisfied	220	9.64
Partially dissatisfied	310	13.58
General	1,258	55.10
Partially satisfied	407	17.83
Very satisfied	88	3.85
**Social capital-trust**		
Not at all	16	0.70
Less	28	1.23
General	619	27.11
More	954	41.79
Extremely	666	29.17
**Social capital-Mutual assistance**		
Not at all	15	0.66
Less	20	0.88
General	371	16.25
More	966	42.31
Extremely	911	39.90
**Social capital-Willingness to participate in community/village affairs**		
Not at all	24	1.05
Less	14	0.61
General	250	10.95
More	741	32.46
Extremely	1,254	54.93
**Social capital-Contact with community/village officials**		
Not at all	88	3.85
Less	71	3.11
General	732	32.06
More	762	33.38
Extremely	630	27.60
**Social capital-Community/village's services**		
Very unfair	92	4.03
Partially unfair	64	2.80
General	500	21.90
Partially fair	906	39.68
Very fair	721	31.58
	**Mean**	**Standard deviation**
COVID-19 preventive behaviors (0–4)	3.49	0.60
Flood risk perception (1–25)	12.30	6.44
Response to flood risk (0–3)	2.52	0.74
Community preparedness for flood risk (0–6)	1.94	1.57
Education years	10.90	2.80

Among the final sample, over 60.71% were male, over 85% were married, and 12.57% were members of the community management committee/village committee. In terms of individual occupation, the majority of respondents (71.92%) worked in-county. Meanwhile, the answers to income satisfaction were measured by a 5-point Likert scale from very dissatisfied to very satisfied, and only 21.68% of respondents were reported to have a satisfied attitude toward their income. Moreover, most subjects (89.18%) had not experienced other disasters in the past, while only 10.82% were covered by agricultural insurance. On average, the participants had at least 10.90 years of education experience.

### Correlation analysis

Table 2 displays the findings of the correlation matrix model, which revealed that COVID-19 preventive behaviors were significantly related to the response against floods, social capital, and community preparedness for flood risk. In detail, those with much higher protective behaviors against floods, social capital, and community preparedness for flood risk were more likely to report higher COVID-19 preventive behavior scores. However, COVID-19 preventive behaviors were found to be uncorrelated with flood risk perception. In addition, there was no significant correlation between flood risk perception and response behaviors, whereas response to flood risk was significantly associated with social capital and community preparedness. Additionally, social capital and community preparedness were found to be related to flood risk perception.

**Table 2:** Correlation analysis (*N* = 2,283).

	**X_1_**	**X_2_**	**X_3_**	**X_4_**	**X_5_**	**X_6_**	**X_7_**	**X_8_**	**X_9_**	**X_10_**	**X_11_**	**X_12_**
X_1_	1.00											
X_2_	0.29^***^	1.00										
X_3_	0.03	−0.02	1.00									
X_4_	0.16^***^	0.15^***^	0.12^***^	1.00								
X_5_	0.18^***^	0.26^***^	0.04^*^	0.48^***^	1.00							
X_6_	0.06^**^	0.01	−0.01	−0.07^***^	−0.05^*^	1.00						
X_7_	−0.03	−0.07^***^	0.06^**^	−0.04	−0.03	−0.05^*^	1.00					
X_8_	0.05^*^	0.02	−0.02	0.23^***^	0.25^***^	−0.08^***^	0.07^***^	1.00				
X_9_	−0.07^**^	−0.10^***^	0.03	0.06^**^	0.04	0.06^**^	0.04	0.14^***^	1.00			
X_10_	0.09^***^	0.07^***^	−0.07^***^	0.12^***^	0.16^***^	−0.04	−0.02	0.13^***^	0.02	1.00		
X_11_	0.05^*^	0.08^***^	−0.02	0.05^*^	0.08^***^	−0.05^*^	−0.01	0.03	−0.00	0.01	1.00	
X_12_	−0.00	−0.04	0.03	0.28^***^	0.17^***^	0.02	−0.03	0.13^***^	0.08^***^	0.12^***^	−0.01	1.00

X_1_-COVID-19 preventive behaviors, X_2_-Response to flood risk, X_3_-Flood risk perception, X_4_-Social capital, X_5_-Community preparedness for flood risk, X_6_-Gender, X_7_-Marital status, X_8_-Member of the community management committee/village committee, X_9_-Workplace, X_10_-Agricultural insurance participation status, X_11_-Disaster experience, X_12_-Satisfaction with income.

* *P* < 0.05,

** *P* < 0.01,

*** *P* < 0.001.

### Linear regression analysis

Linear regression analysis explored the relationships between risk perception, protective behaviors of natural hazards, and COVID-19 preventive behaviors. Correspondingly, Models 1–3 were shown with COVID-19 preventive behaviors as the outcome variable and flood risk perception, response to flood risk, or both as independent variables. Specifically, Model 1 revealed that flood risk perception could positively predict COVID-19 preventive behaviors (beta = 0.04, *P* < 0.05). The response to flood risk was also related to COVID-19 preventive behaviors (beta = 0.28, *P* < 0.001), shown in Model 2. As Model 3 illustrated, flood risk perception (beta = 0.04, *P* < 0.05) and response to flood risk (beta = 0.28, *P* < 0.001) were both significantly associated with COVID-19 preventive behaviors.

All the potential confounding variables were controlled in Models 1–3. In comparison, women and members of the community management committee/village committee were more likely to adopt preventive behaviors against COVID-19. Moreover, those with agricultural insurance and disaster experience reported more COVID-19 preventive behaviors, whereas respondents who chose to work in-county were less inclined to take protective measures against COVID-19. More details are listed in Table 3.

**Table 3:** The relationships among flood risk perception, response behaviors, and COVID-19 preventive behaviors (*N* = 2,283).

**Variable**	**Model 1**	**Model 2**	**Model 3**
	**Beta (*P*-value)**	**Beta (*P*-value)**	**Beta (*P*-value)**
Flood risk perception	0.04^*^	−	0.04^*^
Response to flood risk	−	0.28^***^	0.28^***^
**Gender (Ref: Male)**			
Female	0.08^***^	0.07^***^	0.08^***^
Education years	0.02	−0.01	−0.01
**Marital status (Ref: Unmarried)**			
Married	−0.01	−0.00	−0.00
Windowed/Divorced	−0.03	−0.02	−0.03
**Member of the community management committee/village committee (Ref: No)**			
Yes	0.06^**^	0.05^*^	0.05^*^
**Workplace (Ref: Out-of-county)**			
In-county	−0.08^***^	−0.05^*^	−0.05^**^
**Agricultural insurance participation status (Ref: No)**			
Yes	0.09^***^	0.07^***^	0.07^***^
**Disaster experience (Ref: No)**			
Yes	0.06^**^	0.03	0.03
Satisfaction with income	−0.02	−0.01	−0.01

Beta, standardized coefficient;

* *P* < 0.05,

** *P* < 0.01,

*** *P* < 0.001.

### Moderating effect analysis

**Figure 1:**
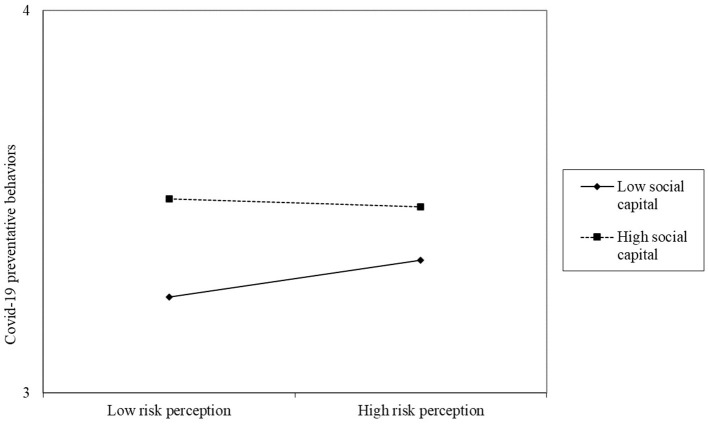
The moderating effects of social capital on the relationship between flood risk perception and COVID-19 preventive behaviors.

**Figure 2:**
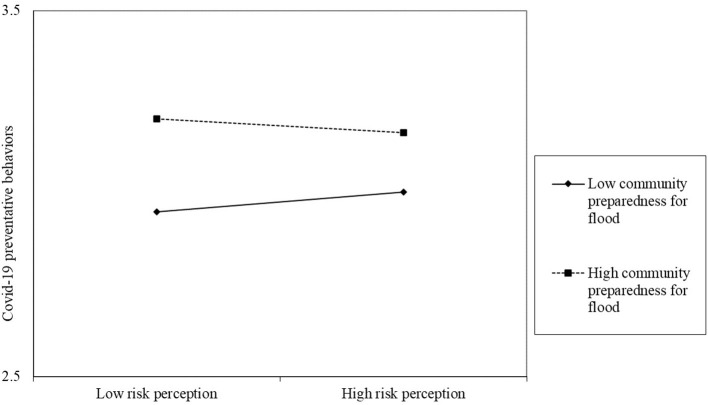
The moderating effects of community preparedness for flood risk on the relationship between flood risk perception and COVID-19 preventive behaviors.

According to the results of the linear regression models, the moderating effect analyses were conducted to examine the roles of social capital and community preparedness for flood risk in the relationship between flood risk perception and COVID-19 preventive behaviors and the relationship between flood response and COVID-19 preventive behaviors. All findings are presented in Table 4, obtained after controlling for confounding variables such as gender, years of education, disaster experience, etc. In particular, Model 4 revealed that social capital significantly modifies the relationship between flood risk perception and COVID-19 preventive behaviors (beta = −0.05, *P* < 0.05). The corresponding diagram of Model 4 is shown in Figure 1, indicating that individuals with higher social capital were more likely to adopt COVID-19 preventive behaviors. Furthermore, with community preparedness for flood risk as the moderating variable, Model 5 was conducted to analyze its role in the relationship between flood risk perception and COVID-19 preventive behaviors. The results showed that the standardized regression coefficient of the interaction terms significantly affects the COVID-19 preventive behaviors (beta = −0.04, *P* < 0.05), which can also be seen in Figure 2. However, at the same time, neither social capital nor community preparedness for flood risk had a moderating effect on the response to flood risk affecting COVID-19 prevention. Specifically, the effect of response to flood risk on COVID-19 preventive behaviors was not moderated by social capital significantly (Beta = 0.01, *P* > 0.01). The moderate effect of community preparedness for flood risk on the above correlation were also not significant (Beta = −0.02, *P* > 0.01).

**Table 4:** The moderating effects of social capital and community preparedness for flood risk (*N* = 2,283).

**Variable**	**Model 4**	**Model 5**	**Model 6**	**Model 7**
	**Beta (*P*-value)**	**Beta (*P*-value)**	**Beta (P-value)**	**Beta (*P*-value)**
Flood risk perception	0.03	0.01	−	−
Response to flood risk	−	−	0.26^***^	0.24^***^
Social capital	0.17^***^	−	0.13^***^	−
Flood risk perception * social capital	−0.05^*^	−	−	−
Response to flood risk * social capital	−	−	0.01	−
Community preparedness for flood risk	−	0.17^***^	−	0.11^***^
Flood risk perception * Community preparedness for flood risk	−	−0.04^*^	−	−
Response to flood risk * Community preparedness for flood risk	−	−	−	−0.02
Potential confounding variables were controlled

Beta, standardized coefficient;

* *P* < 0.05,

*** *P* < 0.001.

## Discussion

In the context of the COVID-19 epidemic, the current study sought to explore the effect of a specific flooding event in China caused by climate change on COVID-19 prevention at the individual level. Meanwhile, the moderating effect of community context, including social capital and community disaster preparedness, on the above relationship has also been examined.

First, flood risk perception could positively predict COVID-19 preventive behaviors, consistent with some disaster-related models, such as PMT (10, 11, 60). As one of the most widely applied disaster prevention decision-making models (61), PMT-related studies posited that individuals might start to feel fearful of the severe damage of potential hazards once they appraise threats, which could motivate them to engage in protective actions (62). Here, individuals exposed to extreme rainfall may simultaneously have a higher awareness of impacts of COVID-19, predicting high-risk perceptions regarding multiple hazards (63), which encouraged the public to engage in disaster reduction activities, such as preventive behaviors against COVID-19. However, the result is inconsistent with a multi-hazard study conducted in Beijing, China, the percentage of individuals more concerned about COVID-19 was reduced by 9.4%, and the likelihood of those wearing masks decreased by 20.6% during heatwaves (64). The differences in the above studies may be due to types of disaster. Compared to the floods experience, individuals exposed to heatwaves were less likely to wear masks in order to avoid potential risks such as sunstroke. In addition, previous studies regarding compound risk of floods and COVID-19 were inclined to focus on the effect of COVID-19 on protective behaviors against floods. For example, a prior study conducted in Kumamoto, Japan indicated that COVID-19 had a major impact on evacuation and volunteerism at the time of the flood. Specifically, individuals perceived the threat of COVID-19 were more likely to hesitated whether to evacuate to the designated evacuation center with various preventive measures or whether to participate in volunteer activities (65). Another study conducted in the US during the hurricane season revealed that COVID-19 risk perception negatively affected response strategies against hurricanes (22). Overall, our findings enrich the research regarding the compound flooding risk in the COVID-19 pandemic. Second, the response to flood risk was positively associated with preventive behaviors against COVID-19. The result indicated that individuals who adopted more flood response measures were more likely to prevent COVID-19 thoroughly. Similarly, PADM with a feedback session indicated that individuals' behavioral decision against hazards would influence their subsequent action decision process and eventually update their behavior (24). Likewise, the response to flood risk could influence individuals' behavioral decision-making process against COVID-19 and prompt them to take positive measures.

Based on the discussion of the correlation between flood risk perception and preventive behaviors against COVID-19, and between response to flooding risk and preventive behaviors against COVID-19, our study suggested that individuals exposed to a specific flooding event in China caused by climate change were more inclined to take measure against COVID-19. The results appeared to contrast with previous studies highlighting that preventive measures against COVID-19 are challenging to continue during the occurrence of natural hazards (66) because COVID-19 virus containment strategies such as social distancing, self-isolation, and regular washing of hands became more difficult to sustain when a natural disaster hit (67). The resurgence of COVID-19 in coastal states of the USA in 2020 was confirmed to be related to the active Atlantic hurricane season (68). By comparison, the current studies on the positive effect of flood risk perception and response on COVID-19 preventive behaviors enrich the existing research on compound risk to a certain extent.

Moreover, social capital was found to moderate the relationship between flood risk perception and COVID-19 preventive behaviors but not the relationship between response to flood risk and preventive behaviors against COVID-19. First, the results suggested that if individuals perceive their social capital in the community they belong to as high, they are more likely to adopt more preventive behaviors in response to COVID-19 even if their threat appraisal of flood risk is lower. The role of social capital in the risk response has been supported in previous studies (69, 70). Specifically, social capital could provide individuals access to various resources in response to hazards, including immediate aid, hazard information, living essentials, and emotional support, since individuals with high social capital are more likely and able to cooperate with others or offer help during disasters (32). In addition, mutual trust could effectively raise awareness of emergency management and volunteer participation, enhancing the community's hazard response capacity (71). The elements of social capital discussed above have also been shown to predict preventive behaviors against COVID-19 effectively (72, 73). Moreover, the current study concluded that social capital could affect COVID-19 preventive behaviors by interacting with flood risk perception but not with the response behaviors of flood risk. The potential reason for the difference may be due to the difference between risk perception and response behaviors. According to existing decision-making models such as PMT and MIV, the process from risk perception to behavior is complex. It can be influenced by various factors, such as social capital. By comparison, individuals' behaviors may be more stable and less likely to change.

Similarly, the interaction between flood risk perception and community disaster preparedness was identified to have a moderating effect on preventive behaviors against COVID-19, which was in line with the conclusion of the health belief model (HBM). In the HBM, individuals' belief in disaster preparedness could be positively associated with individual behaviors against hazards (74). Therefore, those who perceived community disaster preparedness as high were more likely to adopt preventive behaviors against COVID-19 in the current study. A previous study also confirmed a positive interaction between community disaster preparedness and preventive measures at the household/individual level (36). The possible reason for the positive relationship could be that individuals living in communities with good disaster preparedness were more likely to build social networks with others, which is beneficial for individuals to adopt preventive behaviors because they can obtain resources through the social network such as disaster-related information (75). Again, the current study found that community disaster preparedness could influence COVID-19 preventive behaviors through interacting with flood risk perception but not through interacting with response behaviors of flood risk. The difference in risk perception and behavior discussed above could still explain it.

Furthermore, our research also involved other variables potentially associated with preventive behaviors, such as gender, education status, marital status, community management committee/village committee membership, workplace, insurance, disaster experience, and income. First, the results indicated that females were more likely to implement behavioral changes in response to COVID-19, which is in line with a previous study (76). Contrary to the existing evidence, it was impossible to affirm that higher education could predict better preventive behaviors against COVID-19 (77). Regarding marital status differences, our results showed no significant difference among marital statuses with preventive behaviors against COVID-19. These results appeared to contrast with the research conducted by Li and his colleagues, who argued that marital status was significantly associated with preventive behaviors (78). In addition, as reported in the literature conducted among members of the Communist Party of China (79), members of the community management committee/village committee were more likely to adopt preventive behaviors against hazards. In terms of workplace differences, our research found significant differences among workplaces with COVID-19 preventive behaviors, consistent with a previous study (74). Insurance was a protective factor for preventive behaviors against COVID-19 in the current study. Specifically, those insured were more likely to show high-risk perception, prompting them to take protective measures in response to COVID-19 (80). In addition, there was no relationship between income and COVID-19 preventive behaviors, whereas Maria and colleagues found a correlation between those two variables (81).

However, some limitations need to be acknowledged. The research was limited by a cross-sectional design, and the results cannot be used to draw causal inference conclusions. Additionally, although we have considered social capital and community disaster preparedness, behavior decision-making is complex, and we cannot enumerate every relevant variable.

## Conclusion

The current study conducted in China provided insight into how the public changed their behavior in response to COVID-19 when sudden flooding struck. We found that after floods, individuals' risk perception and response behaviors significantly correlated with their prevention behaviors against COVID-19. Furthermore, community disaster preparedness and social capital moderated the above relationships to a certain extent. The findings can guide risk communication under the compound risk scenario and prevent risky public behavior under the consistent presence of COVID-19 in the community. In addition, community disaster risk reduction activities must be integrated into regular social governance, focusing on vulnerable people who are not closely connected to the community.

## Data availability statement

The data analyzed in this study is subject to the following licenses/restrictions: Data is not publicly available. Requests to access these datasets should be directed to qz@bnu.edu.cn.

## Ethics statement

The studies involving human participants were reviewed and approved by the School of Social Development and Public Policy of Beijing Normal University. The patients/participants provided their written informed consent to participate in this study.

## Author contributions

CL contributed to the conceptualization, methodology, investigation, data curation, formal analysis, and writing-original draft and editing. QL contributed to the conceptualization, methodology, investigation, formal analysis, and writing—review and editing. QZ contributed to the conceptualization, methodology, investigation, writing—review and editing, and project administration. All authors contributed to the article and approved the submitted version.

## Funding

China Foundation for Rural Development provided the financial supports for the field study of this study.

## Conflict of interest

The authors declare that the research was conducted in the absence of any commercial or financial relationships that could be construed as a potential conflict of interest.

## Publisher's note

All claims expressed in this article are solely those of the authors and do not necessarily represent those of their affiliated organizations, or those of the publisher, the editors and the reviewers. Any product that may be evaluated in this article, or claim that may be made by its manufacturer, is not guaranteed or endorsed by the publisher.

## Footnotes

^1^World Health Organization. WHO Coronavirus (COVID-19) Dashboard. https://covid19.who.int/ [Accessed July 22, 2022].
